# Epigenetic Aging in Early Life: Role of Maternal and Early Childhood Nutrition

**DOI:** 10.1007/s13668-022-00402-7

**Published:** 2022-02-22

**Authors:** Nicholas A. Koemel, Michael R. Skilton

**Affiliations:** 1 The Boden Initiative, Charles Perkins Centre, The University of Sydney, Sydney, Australia; 2 Sydney Medical School, Faculty of Medicine and Health, The University of Sydney, Sydney, Australia; 3 Sydney Institute for Women, Children and Their Families, Sydney Local Health District, Sydney, Australia

**Keywords:** Maternal diet, Epigenetics, Early life, Childhood nutrition, Aging, Developmental Origins of Health and Disease (DOHaD)

## Abstract

***Purpose of Review*:**

Early life presents a pivotal period during which nutritional exposures are more likely to cause epigenetic modifications, which may impact an individual’s health during adulthood. This article reviews the current evidence regarding maternal and early childhood nutritional exposures and their role in epigenetic aging.

***Recent Findings*:**

Maternal and early life consumption of diets higher in fiber, antioxidants, polyphenols, B vitamins, vitamin D, and ω-3 fatty acids is associated with slower epigenetic aging. Conversely, diets higher in glycemic load, fat, saturated fat, and ω-6 fatty acids demonstrate a positive association with epigenetic aging.

***Summary*:**

Maternal and early life nutrition directly and indirectly influences epigenetic aging via changes in one-carbon metabolism, cardiometabolic health, and the microbiome. Clinical trials are warranted to determine the specific foods, dietary patterns, and dietary supplements that will normalize or lower epigenetic aging across the life course.

## Introduction

The Barker hypothesis, now more frequently referred to as the Developmental Origins of Health and Disease (DOHaD), originated from epidemiological evidence published in the 1980s demonstrating a clear inverse association of newborn birth weight with death from cardiovascular diseases in adulthood [1]. In the three and a half decades since the publication of these initial observations, research in this field has generated a large body of evidence regarding the long-term effects of early life exposures, including the role of maternal diet in fetal development and health and disease in later life [2]. Fetal epigenetic modifications may play an important role as a mechanism that links maternal nutrition with both fetal development and long-term health outcomes for the affected fetus.

**Fig. 1:**
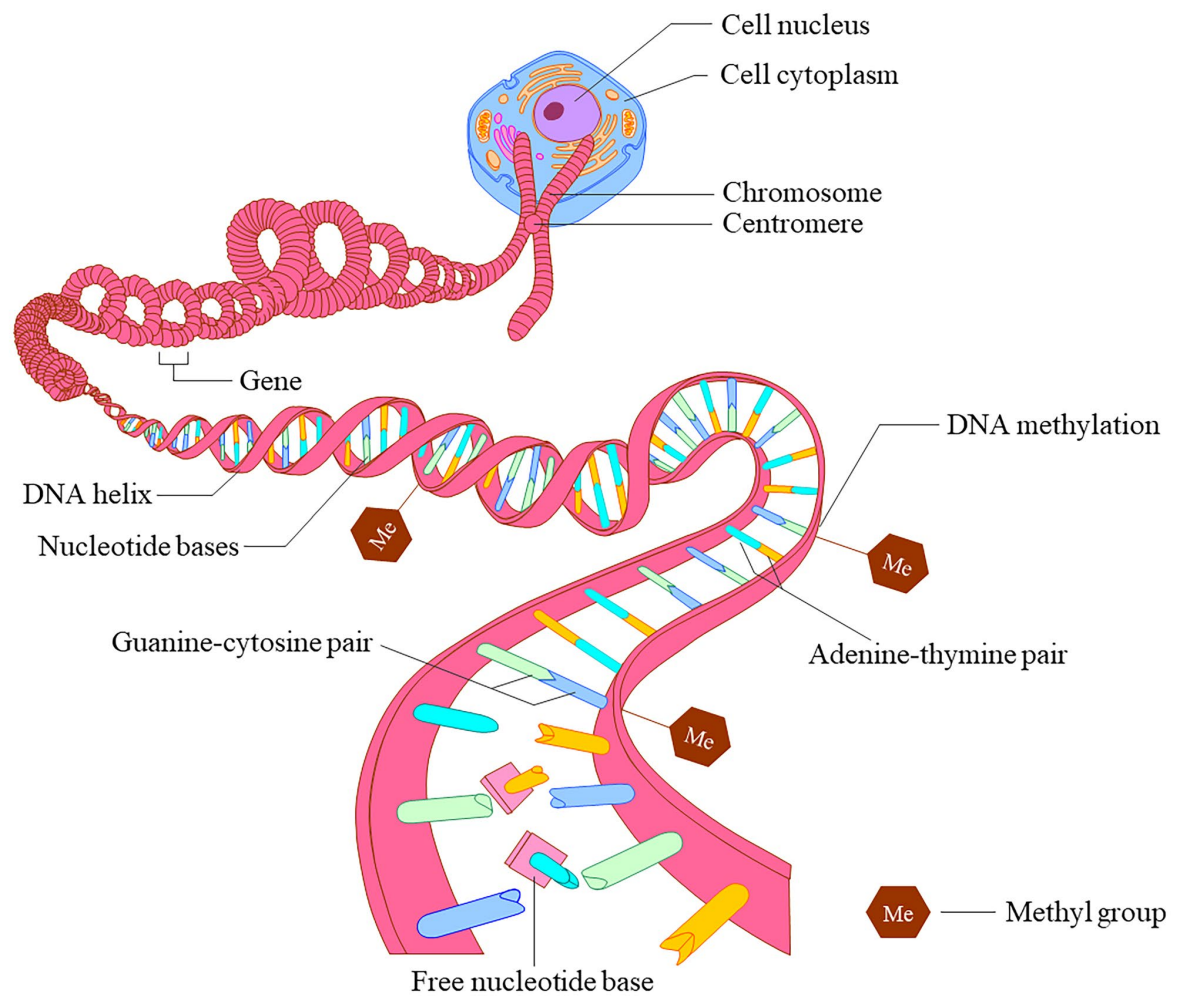
DNA methylation

Epigenetics involves a wide range of heritable biological changes that alter gene expression or activity without directly manipulating the DNA sequence [3]. Fundamental epigenetic changes include histone modifications and DNA methylation [4]. Histone modifications typically involve the addition of an acetyl unit by histone acetyltransferases, which can lead to post-transcriptional changes [4]. DNA methylation often occurs at regions of DNA where cytosine is immediately followed by guanine, known as CpG sites (Fig. 1) [5]. There are numerous CpG sites that when either hyper or hypo-methylated can affect gene expression. Individually or collectively, the methylation status of many of these sites is associated with markers of metabolic health [6]. Nutritional exposures play a vital role in regulating epigenetic processes by directly providing necessary substrates and indirectly by changes in metabolism. Fetal and early life presents a period where the human epigenome has a high degree of plasticity and is susceptible to external exposures. As such, maternal and early childhood nutrition is likely an important exposure driving epigenetic programming in the young [2].

A growing body of literature focuses on epigenetic age derived from epigenetic “clocks,” equations derived from the methylation status of a number of specific age-related CpG sites [7]. In general, epigenetic age acceleration reflects epigenetic age above or below chronological age, and as such is a measure of biological aging. A recent review concluded that epigenetic clocks provide a better indicator of biological aging than other markers, including proteomic predictors, transcriptomic predictors, telomere length, metabolomic predictors, and biomarkers [8]. Poor metabolic health, including oxidative stress [9] and chronic inflammation [10], is an important risk factor for epigenetic age acceleration [11]. Importantly, epigenetic aging is associated with future incidence of cardiovascular disease [12], cancer [13], diabetes [14], and all-cause mortality [15], independent of chronological aging. These epigenetic clocks are now being applied to study the influences of nutrition, lifestyle, and environmental factors on the aging process [16].

This narrative review identifies, highlights, and discusses the most recent evidence and key concepts relating to maternal and early life nutrition and epigenetic aging, and proposes future research priorities.

## Findings

### Maternal Under- and Overnutrition

**Fig. 2:**
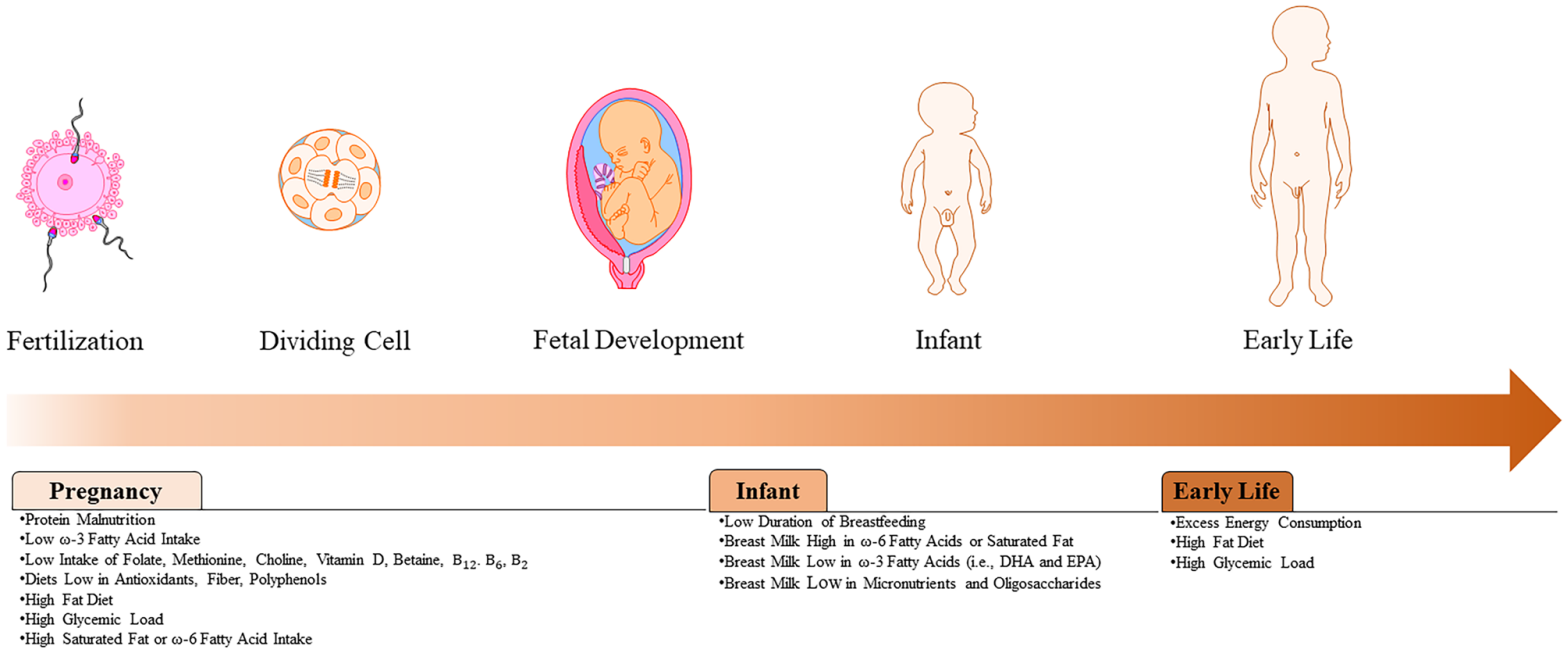
Nutritional characteristics during pregnancy and early life that are linked with increased epigenetic aging in the offspring

Epidemiological data from extreme maternal dietary restriction, such as the Dutch Hunger famine [17], provide proof-of-principle that severe maternal caloric restriction is associated with a higher incidence of metabolic disorders in the offspring. This may be partially related to the inadequacy of macro- and micronutrients (Fig. 2) which provide substrates that are important for healthy fetal development, including protein, folate, choline, betaine, vitamin B_12_, vitamin B_6_, vitamin B_2_, antioxidants, and bioactive compounds. Protein malnutrition has been widely studied given the role of amino acids in the healthy development of the vital organs. Animal models show decreased pancreatic β-cell mass in the offspring of pregnant dams consuming a low-protein diet [18], which is sustained into adulthood in addition to hyperinsulinemia, and reduced insulin signaling protein expression [19].

These nutrients not only play a role in development but also are needed for the regulation of oxidative stress, inflammation, cellular division, and an adequate supply of the one-carbon units required for DNA methylation [20]. Maternal undernutrition may affect the fetal epigenome through reduced intake and bioavailability of key micronutrients involved in the one-carbon metabolism such as folate, methionine, choline, betaine, vitamin B_12_, vitamin B_6_, vitamin B_2_, antioxidants, and other bioactive compounds [21, 22], discussed in detail later in this review.

At the other end of the spectrum, maternal overnutrition also appears to contribute to epigenetic modifications. Extreme maternal overnutrition can lead to a variety of maternal metabolic factors, such as obesity, hyperlipidemia, poor glycemic control, hypertension, and low-grade inflammation, all of which may modify the offspring’s epigenome [23–25]. For example, in a recent meta-analysis of 19 cohorts comprising 9,340 mother-newborn pairs, maternal pre-pregnancy body mass index was associated with DNA methylation variation at 104 CpG sites [26]. However, maternal obesity and excessive gestational weight gain appear to have mixed associations with epigenetic aging of the offspring [27, 28••, 29], possibly due to differences in newborn body weight [30].

### Impaired Fetal Growth

Maternal nutrition provides the fetal nutrient supply necessary for growth and development during pregnancy. Both under- and overnutrition can disrupt nutrient supply causing impaired fetal development [31]. However, placental function and development are likely more substantial contributors to fetal growth for the majority of births in developed nations where severe undernutrition is rare [32]. Newborns who experienced impaired growth often show early signs of disrupted glucose metabolism, hypothalamus–pituitary–adrenal axis, and vascular function, predisposing them to cardiovascular and metabolic disorders in later life [33]. Whether birth weight itself is associated with epigenetic age acceleration is less clear. Recent research has demonstrated that newborns of a preeclamptic pregnancy [34] or low birth weight [35, 36] show accelerated placental epigenetic aging. In contrast, Phang et al. found that neither birth weight nor body fatness is associated with epigenetic age acceleration in newborns [28••]. In a small study, 30–35-year-old males who had been born with extremely low birth weight had accelerated epigenetic aging compared to their peers born with normal birth weight [37]. Taken together, these associations suggest links between maternal–fetal wellbeing and offspring epigenetic aging, although the finer detail of these links requires further description.

### Maternal Dietary Macronutrients

Dietary macronutrients, carbohydrate, protein, and fat are the principal sources of dietary energy. Macronutrients may influence health and disease outcomes through regulation of energy intake, but there is a large body of evidence indicating their associations with disease outcomes independent of caloric intake (i.e., within the context of an isocaloric diet). The role of maternal macronutrient intake and offspring epigenetic aging has been increasingly explored.

#### Carbohydrates

There is an inverse association between maternal carbohydrate intake and epigenetic age acceleration in newborns [28••]. However, carbohydrates are derived from various sources and can differ in quality. Glycemic index (GI), a measure of the glucose-raising effect of foods, is considered a marker of carbohydrate quality. Secondary and post hoc analyses of several recent trials of low GI diets during pregnancy provide some support for a detrimental effect of GI on epigenetic modifications related to insulin regulation in both placental [38] and newborn tissues [39]. However, the relationship with carbohydrates remains complex and difficult to isolate given that carbohydrate is typically the primary source of dietary energy, and as such, the intake of many micronutrients is associated with carbohydrate intake. Similarly, other nutritional elements such as fiber often co-inhabit with minimally processed carbohydrate-rich foods.

#### Protein

Maternal protein intake does not appear to be meaningfully associated with offspring epigenetic aging [28••], although evidence remains limited. In animal models, inadequate maternal protein intake worsens metabolic and cardiovascular health outcomes [40]. During pregnancy, low dietary protein may lead to an insufficient supply of amino acids necessary for one-carbon metabolism (i.e., methionine), which is integral to many epigenetic processes. Other evidence points toward the role of protein-restricted pregnancies in regulating peroxisome proliferator–activated receptors (PPARs) [41]. PPARs are nuclear receptor proteins involved in the regulation of gene expression by conducting an essential role in many cellular processes including cell differentiation, inflammation, and metabolism [42]. In animal models with protein-restricted pregnancies, offspring appear to have reduced expression of PPAR-α, which has been implicated in the maintenance of energy metabolism and oxidative stress, potentially contributing to the aging process [43].

#### Fat

A maternal high-fat diet is positively associated with newborn epigenetic aging [28••]. It has been proposed that this may be at least partially related to elevated serum lipid levels, insulin resistance, oxidative stress, and systemic inflammation during pregnancy [44], and involve the gut microbiome. For example, a maternal high-fat diet during pregnancy disrupts the maternal gut microbiome and increases lipid accumulation in the offspring’s liver [45]. These changes to the maternal gut microbiome could be transgenerational and persist in the offspring beyond the immediate postnatal period, with a maternal high-fat diet leading to infant microbial dysbiosis up to 4–6 weeks after birth in animal models [46]. These changes in microbiome health could lead to higher offspring exposure to toxins and pro-inflammatory markers involved in epigenetic aging.

### Maternal Dietary Fatty Acids

Specific dietary fatty acids of varying chain length and saturation exhibit different associations with offspring epigenetic modifications [47]. Maternal dietary saturated fat, ω-6 polyunsaturated fat, and trans-saturated fat have positive associations with age-related inflammatory markers, lipid metabolism, and disrupted cellular functionality [47]. Conversely, ω-3 polyunsaturated fatty acid supplementation has been widely recognized for its anti-inflammatory and epigenetic effects [48]. These studies of epigenetic related mechanisms support a recent study of maternal diet and offspring epigenetic age by Phang et al., in which there was a strong positive association of maternal saturated fat and palmitoleic acid intake with epigenetic aging in the newborn offspring, while ω-3 polyunsaturated fat intake was inversely associated with newborn epigenetic aging [28••]. Koemel et al. demonstrated that the composition of maternal dietary fatty acids is associated with newborn epigenetic aging such that the association of each fatty acid class with epigenetic age acceleration is dependent upon the levels of other fatty acids [49•]. For example, ω-3 polyunsaturated fats appear to only provide a protective effect against epigenetic aging in offspring when maternal intake of saturated fat or palmitoleic acid is also high. This interaction might be due to the differential epigenetic effects of specific fatty acids that could not be revealed when assessed individually. These findings suggest that associations of maternal fatty acids with offspring epigenetics may be more complex than previously described.

### The Role of Micronutrients and Fiber

Micronutrients play a crucial role in fetal development and maintaining metabolic homeostasis during pregnancy. In the context of epigenetic modifications, micronutrients involved in one-carbon metabolism such as folate, choline, betaine, and methionine act as methyl donors, while vitamin B_12_, vitamin B_6_, and vitamin B_2_ act as cofactors involved in the transfer of methyl units [50]. Maternal dietary deficiency in these nutrients can result in changes to DNA methylation in the offspring [51, 52], and epigenetic aging. Animal studies show that supplementation with folate or B_12_ can reduce epigenetic aging in adults [53]. Within the context of maternal diet, a mother’s deficiency in these methyl donors can lead to low levels of one-carbon units needed for DNA methylation and elevated detrimental byproducts of disrupted one-carbon metabolism such as homocysteine which are associated with accelerated epigenetic aging in offspring [54].

Vitamin D is widely accepted to play a role in the development of human tissues, gene expression [55], and global DNA methylation [56]. Evidence from an observational study in humans supports an inverse association between maternal vitamin D consumption and epigenetic aging of newborns [28••]. In a recent post hoc analysis of a small randomized trial of ninety-two multi-ethnic pregnant women, offspring epigenetic age acceleration was not affected by 4000 IU/day of vitamin D3 compared to placebo [57]. There was, however, a beneficial effect of the vitamin D3 supplementation, evidenced by reduced offspring epigenetic aging, in the offspring of African American women when assessed by both Knight’s epigenetic clock (*β* =  −0.89, *p* = 0.047) and Bohlin’s epigenetic clock (*β* =  −0.71, *p* = 0.005). Confirmation of these putative protective effects of maternal vitamin D3 supplementation as prespecified outcomes in larger experimental trials should be considered a priority.

Other bioactive compounds such as antioxidants and polyphenols play a crucial role in reducing systemic inflammation and maintaining reactive oxygen species homeostasis [22]. Experimental animal models demonstrate that supplementation of polyphenol compounds can affect the regulation of DNA methylation by altering the activity of non-coding RNAs, histone deacetylases, and DNA methyltransferase [58]. In animal models, supplementation of bioactive polyphenol-rich food components, such as resveratrol, has been shown to partially protect against the deleterious epigenetic metabolic programming of mothers consuming a low-protein diet [59]. Nonetheless, no studies have thus far explored the associations of maternal intake of these bioactive components on offspring epigenetic aging in humans.

Maternal dietary fiber also has a beneficial association with offspring metabolic health [60]. This may at least partially be mediated by the vital role of fiber in the maintenance and colonization of the maternal microbiome, which in turn contributes to the colonization of the offspring microbiome. Maternal microbial health during pregnancy is a known regulator of inflammation, immune function, and the production of short-chain fatty acids [61]. Specific short-chain fatty acids such as butyrate can directly influence epigenetic modifications by acting as a histone deacetylase inhibitor [62]. In support of this, a recent trial of a probiotic intervention during pregnancy found decreased DNA methylation in obesity and weight gain–related genes in both the mother and child [63]. Other evidence from animal models points toward the metabolic health impact of prenatal colonization such as improved glycemic control, reduced inflammation, and lower risk of obesity [64, 65]. That said, despite increasing discussion of a link between maternal microbiome health and newborn epigenetic aging [66], limited research has directly informed this topic.

### Foods, Dietary Patterns, and Dietary Quality

Food-level analyses, dietary patterns, and measures of dietary quality present alternative frameworks through which to assess nutritional characteristics. In adults, food groups such as fruits and nuts, and foods rich in whole grains, are inversely associated with epigenetic age acceleration [67]. In contrast, red meat consumption is directly associated with epigenetic age acceleration. From a more encompassing dietary pattern perspective, adherence to more elements of the Dietary Approaches to Stop Hypertension (DASH) diet was associated with reduced epigenetic aging [68]. Higher quality diets are more likely to provide adequate amounts of micronutrients, antioxidants, and polyphenols while concomitantly limiting potentially metabolically deleterious dietary components such as saturated fat and refined sugars. As such, determining whether maternal dietary patterns are associated with offspring epigenetic age acceleration, and whether any such associations are independent of specific nutrient characteristics, should be considered a priority.

### Neonatal Nutrition

Human breast milk or formula is the primary source of nutrients for neonates and plays a vital role in the modification of the epigenome during infancy [69]. Longer duration of breastfeeding has been linked with epigenetic modification to obesity-related genes such as the leptin gene, consistent with the well-described association of breastfeeding duration with lower risk of obesity in later life [70]. Recently, differences in epigenome-wide changes from birth to 10 years were examined in those who were exclusively breastfed or exclusively formula-fed as infants for at least 3 months, with 87 CpG sites showing differences in DNA methylation [71•]. Interestingly, the exclusively breastfed neonates showed positive methylation for genes involved in organelle biogenesis (TTC30B), DNA binding transcription factors (SOX-1), ligase activity (RNF220), and the production of microRNAs (MIR658). These differences in methylation patterns may lead to changes in cellular functions involved in epigenetic regulation. However, to our knowledge, no CpG sites identified in this study were related to metabolic health or epigenetic clocks.

The difference in total caloric intake between formula and breastfed infants has been proposed as an essential aspect underlying associations of infant feeding status with longer-term health and disease outcomes, particularly obesity-related outcomes. However, the composition of formula and breast milk may also contribute to their short and long-term effects on health and disease. Breast milk fatty acid composition has been examined as a possible pathway for newborn epigenetic modifications [72], with a focus on the maternal intake of long-chain ω-3 polyunsaturated fatty acids, given that the concentration of these fatty acids in breast milk is at least partially linked to maternal intake [73] and that they play a role in early childhood development [74]. The human body is unable to synthesize ω-3 fatty acids endogenously and, as such, is reliant on dietary intake or endogenous conversion from shorter-chain ω-3 fatty acids for maintaining sufficient levels for healthy cellular and tissue function. Thus, the maternal diet can act as a dominant factor for affecting the bioavailability of these nutrients in breastfed infants. Other fatty acids such as ω-6 fatty acids compete for elongase and desaturase enzymes which can impair the endogenous conversion of short-chain ω-3 fatty acids to the long-chain ω-3 fatty acids required for healthy development [75]. Human studies reveal that maternal intakes of ω-6 and ω-3 fatty acids during lactation both directly affect fatty acid composition in breast milk [74]. In general, a higher ratio of ω-6:ω-3 fatty acids can lead to higher levels of inflammation and expression of obesity-associated genes [75]. Further investigation is needed to explore the association of ω-6:ω-3 fatty acid concentration with epigenetic clocks and methylation of age-related genes in neonates.

Breastfeeding seeds the natural colonization of offspring gut microbiota [76]. This may relate to oligosaccharides in breast milk, which provide a substrate to help promote a healthy functioning microbiome, and more directly the limited quantity of microbes in breast milk [77]. The development and maintenance of a healthy microbiome in the neonatal period can shape the epigenome by producing metabolites involved in mediating gene expression and epigenetic processes [78]. However, limited evidence exists regarding the translation of neonatal microbial health with epigenetic aging.

There has been only one study that has directly examined the association of breastfeeding with early life epigenetic aging. Simpkin et al. studied DNA methylation profiles from 1018 mother–child pairs in the Avon Longitudinal Study of Parents and Children [79], with epigenetic age measured at birth, 7, and 17 years of age. There were no significant differences in epigenetic age during childhood or adolescence for those reported as breastfed. This study did not examine breastfeeding duration or breast milk composition, which may have influenced these findings and warrants more detailed investigation.

### Breast Milk Micronutrients and Bioactive Components

Human breast milk also supplies the micronutrients necessary for development during the neonatal period. In a recent systematic review of 59 observational and 43 intervention studies, maternal dietary intake of fat-soluble vitamins, including vitamin D, and water-soluble vitamins, including vitamin B_12_, was associated with their content in breast milk [80]. Vitamin B_12_ is an essential regulator of one-carbon metabolism and in utero epigenetic modifications. Postnatal vitamin D supplementation is linked with epigenetic changes to gene clusters involved in cell migration and cellular membrane function in human infants [81]. No studies have explored the relationship between maternal breast milk micronutrient content with epigenetic age.

Breast milk also contains various bioactive components, including immunoglobulins, hormones, cytokines, and anti-inflammatory proteins such as lactoferrin [82]. These components play an essential role in the regulation of epigenetic-related factors such as cell growth, immune function, and inflammation [82]. Furthermore, differences in breast milk micronutrient and bioactive content by maternal diet quality may provide an additional avenue of epigenetic influence during the neonatal period. Any such effects of breast milk composition would be unlikely to change public health advice concerning breastfeeding, given that breastfeeding is promoted for a wide variety of beneficial health outcomes for mother and child. However, such evidence could inform postnatal maternal supplementation and dietary strategies to optimize offspring biological aging.

### Early Childhood Nutrition

The NU-AGE study has demonstrated that a Mediterranean dietary pattern intervention was associated with reduced epigenetic aging in healthy adults aged 65–72 [83]. Moreover, interventions combining diet advice, exercise, and lifestyle changes have the ability to reverse epigenetic aging in healthy adult males aged 50–72 (*n* = 43) [84•]. Specifically, the dietary advice included consumption of liver, eggs, dark leafy greens, cruciferous vegetables, beets, low glycemic fruit, seeds, tea, prebiotics, and probiotics while avoiding added sugar, dairy, grains, legumes, and beans. After an 8-week intervention, the treatment group was associated with a 3.23-year decrease in epigenetic age compared to the control group.

Given this evidence that nutrition in adulthood can modify epigenetic aging, it is reasonable to propose that dietary nutrition in childhood, beyond infancy, may also affect epigenetic properties, although there is little direct evidence to inform as to whether this is the case. A recent study assessing methyl donor and cofactor nutrient intakes during the first 2–3 years of life revealed no significant relationship with global DNA methylation at 4 years of age [85]. Conversely, dietary intake of methyl donors appears to be beneficially associated with methylation of CpG sites linked to inflammation in children with asthma [86]. This variation in the effects of nutrient supplementation may be partially explained by the wide degree of environmental, psychological, and social exposures that vary across children, or the distinct differences in methylation outcomes [87, 88].

### Developmental Characteristics and Epigenetic Aging

**Fig. 3:**
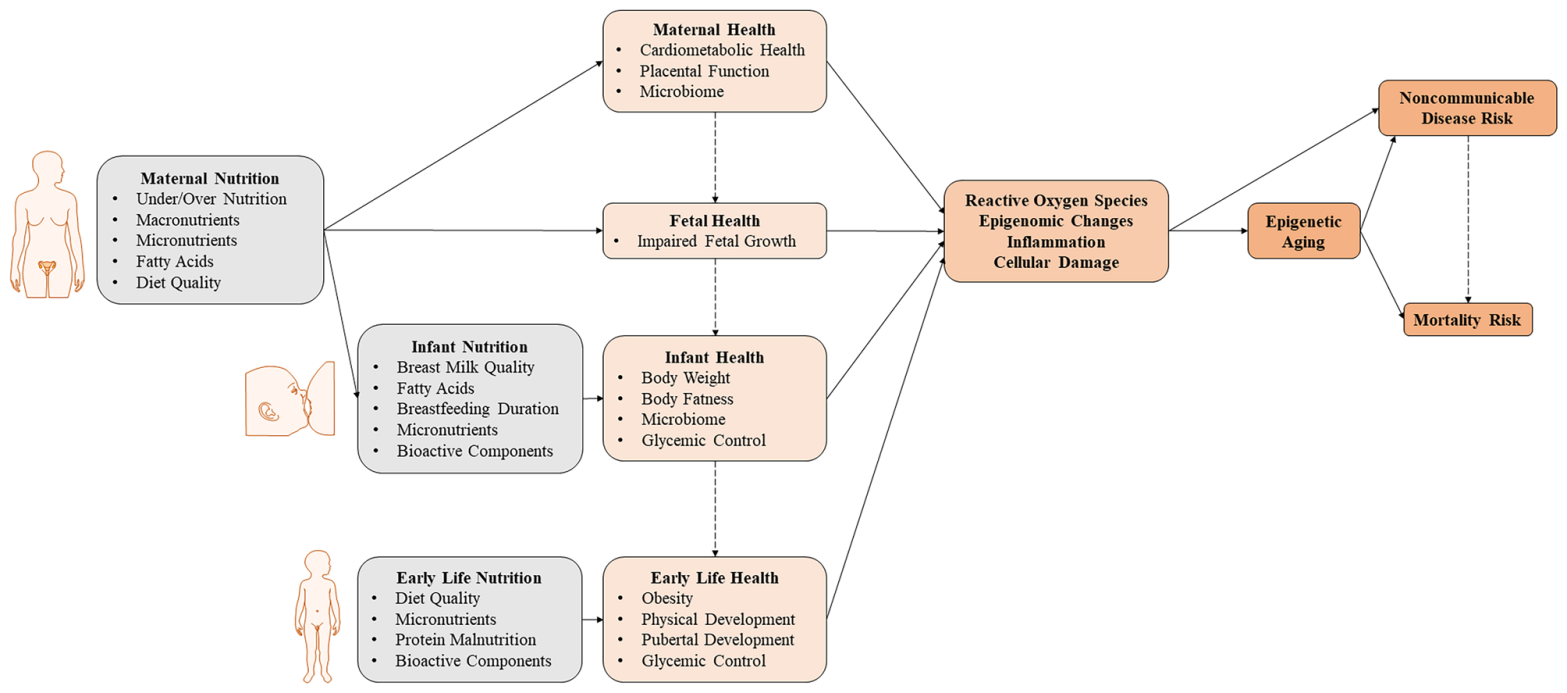
Schematic diagram of the proposed intersection between maternal and early life nutrition, health, and epigenetic aging as pathways to long-term risk of non-communicable diseases

There are numerous causal pathways linking maternal, neonatal, and early life nutrition with markers of health that may ultimately impact epigenetic aging (Fig. 3). A recent study of physical development characteristics with epigenetic aging in 1018 children revealed a positive association of height and fat mass during childhood with epigenetic age acceleration [89]. Specifically, epigenetic age acceleration at birth was positively associated with a higher average fat mass from birth to 17 years of age, and height was positively associated with epigenetic aging at age 7. In a separate study of adolescents, epigenetic aging is related to a higher body mass index, insulin resistance, and pro-inflammatory markers [90•]. Ultimately, these developmental characteristics may help inform the potential links between epigenetic aging, anthropometry and development, and the risk of non-communicable diseases later in adulthood.

## Future Directions

In this review, we have outlined the emerging links between maternal and early life nutrition with epigenetic aging. The broad picture of whether epigenetic aging mechanistically underpins the complex intersection between maternal diet, fetal and infant development, and long-term risk of non-communicable diseases remains a challenging domain. While there is evidence of specific nutrients in the maternal diet being associated with or affecting offspring epigenetic aging, the mechanistic links that are involved remain poorly described. These mechanistic links may involve one-carbon metabolism and the maternal gut microbiome; however, the intersection between maternal diet, early life nutrition, and both the maternal and neonatal gut microbiome is complex. Several potential avenues have yet to be fully elucidated, including the roles of intestinal permeability and microbiome dysbiosis throughout pregnancy. Understanding how the maternal diet-gut axis is affected by a range of nutrients, foods, and dietary patterns, and how these subsequently affect epigenetic aging, should inform future dietary guidelines for the young, with a focus on reducing biological aging to prevent non-communicable diseases.

Despite the relationship between maternal diet and breast milk composition, there is currently a dearth of information regarding the role of maternal diet during lactation and offspring epigenetic aging. Specifically, research regarding maternal micronutrients, antioxidants, and polyphenol intake as it relates to offspring epigenetic aging is greatly needed. This will inform any putative roles of maternal diet, maternal supplementation during lactation, or neonatal supplementation, as plausible pathways to healthy epigenetic programming in high-risk populations.

## Conclusions

Epigenetic aging in adults has a well-demonstrated relationship with non-communicable diseases, cause-specific mortality, and all-cause mortality. Currently, there is an apparent link between maternal and early life nutrition and the epigenetic aging of the offspring, albeit based on an emerging evidence base. A broad range of research is needed to explore these associations throughout early life development, including the determination of causality, underlying mechanisms, and long-term health and disease outcomes.

## References

[1] Barker DJ, Osmond C (1986). Infant mortality childhood nutrition and ischaemic heart disease in England and Wales. Lancet.

[2] Lillycrop KA, Burdge GC (2015). Maternal diet as a modifier of offspring epigenetics. J Dev Orig Health Dis.

[3] Portela A, Esteller M (2010). Epigenetic modifications and human disease. Nat Biotechnol.

[4] Handel AE, Ebers GC, Ramagopalan SV (2010). Epigenetics: molecular mechanisms and implications for disease. Trends Mol Med.

[5] Jones PA, Takai D (2001). The role of DNA methylation in mammalian epigenetics. Science.

[6] Sebert S, Sharkey D, Budge H, Symonds ME (2011). The early programming of metabolic health: is epigenetic setting the missing link?. Am J Clin Nutr.

[7] Pal S, Tyler JK (2016). Epigenetics and aging. Sci Adv..

[8] Jylhävä J, Pedersen NL, Hägg S (2017). Biological age predictors. EBioMedicine.

[9] Srivas S, Baghel MS, Singh P, Thakur MK, Rath PC (2019). Neurodegeneration during aging: the role of oxidative stress through epigenetic modifications. Models molecules and mechanisms in biogerontology: physiological abnormalities diseases and interventions.

[10] Hartnett L, Egan LJ (2012). Inflammation DNA methylation and colitis-associated cancer. Carcinogenesis.

[11] Nannini DR, Joyce BT, Zheng Y, Gao T, Liu L, Yoon G (2019). Epigenetic age acceleration and metabolic syndrome in the coronary artery risk development in young adults study. Clin Epigenetics.

[12] Joyce B, Gao T, Zheng Y, Ma J, Hwang S-J, Liu L (2021). Epigenetic age acceleration reflects long-term cardiovascular health. Circ Res.

[13] Dugué PA, Bassett JK, Joo JE, Jung CH, Ming Wong E, Moreno-Betancur M (2018). DNA methylation-based biological aging and cancer risk and survival: pooled analysis of seven prospective studies. Int J Cancer.

[14] Ling C, Rönn T (2019). Epigenetics in human obesity and type 2 diabetes. Cell Metab.

[15] Marioni RE, Shah S, McRae AF, Chen BH, Colicino E, Harris SE (2015). DNA methylation age of blood predicts all-cause mortality in later life. Genome Biol.

[16] Liu Z, Leung D, Thrush K, Zhao W, Ratliff S, Tanaka T (2020). Underlying features of epigenetic aging clocks in vivo and in vitro. Aging Cell..

[17] Roseboom TJ, Van Der Meulen JH, Ravelli AC, Osmond C, Barker DJ, Bleker OP (2001). Effects of prenatal exposure to the Dutch famine on adult disease in later life: an overview. Twin Res Hum Genet.

[18] Su Y, Jiang X, Li Y, Li F, Cheng Y, Peng Y (2016). Maternal low protein isocaloric diet suppresses pancreatic β-cell proliferation in mouse offspring via miR-15b. Endocrinology.

[19] Fernandez-Twinn D, Wayman A, Ekizoglou S, Martin M, Hales C, Ozanne S (2005). Maternal protein restriction leads to hyperinsulinemia and reduced insulin-signaling protein expression in 21-mo-old female rat offspring. Am J Physiol Regul Integr Comp Physiol.

[20] Jankovic-Karasoulos T, Furness DL, Leemaqz SY, Dekker GA, Grzeskowiak LE, Grieger JA (2021). Maternal folate, one-carbon metabolism and pregnancy outcomes. Matern Child Nutr.

[21] Cai S, Quan S, Yang G, Ye Q, Chen M, Yu H (2021). One carbon metabolism and mammalian pregnancy outcomes. Mol Nutr Food Res.

[22] Silva LBAR, Pinheiro-Castro N, Novaes GM, Pascoal GDFL, Ong TP (2019). Bioactive food compounds, epigenetics and chronic disease prevention: focus on early-life interventions with polyphenols. Food Res Int.

[23] Heerwagen MJ, Miller MR, Barbour LA, Friedman JE (2010). Maternal obesity and fetal metabolic programming: a fertile epigenetic soil. Am J Physiol Regul Integr Comp Physiol.

[24] Shrestha D, Workalemahu T, Tekola-Ayele F (2019). Maternal dyslipidemia during early pregnancy and epigenetic ageing of the placenta. Epigenetics.

[25] Shiau S, Wang L, Liu H, Zheng Y, Drong A, Joyce BT (2021). Prenatal gestational diabetes mellitus exposure and accelerated offspring DNA methylation age in early childhood. Epigenetics.

[26] Sharp GC, Salas LA, Monnereau C, Allard C, Yousefi P, Everson TM (2017). Maternal BMI at the start of pregnancy and offspring epigenome-wide DNA methylation: findings from the pregnancy and childhood epigenetics (PACE) consortium. Hum Mol Genet.

[27] Workalemahu T, Shrestha D, Tajuddin SM, Tekola-Ayele F (2021). Maternal cardiometabolic factors and genetic ancestry influence epigenetic aging of the placenta. J Dev Orig Health Dis.

[28] •• Phang M, Ross J, Raythatha JH, Dissanayake HU, McMullan RL, Kong Y, et al. Epigenetic aging in newborns: role of maternal diet. Am J Clin Nutr. 2020;111(3):555–61. **This study revealed that a high-fat maternal diet and those higher in saturated fat or palmitoleic acid were associated with higher epigenetic age acceleration in newborns. This study also showed lower epigenetic age acceleration with maternal vitamin D supplementation.** 10.1093/ajcn/nqz326 31942922

[29] Khouja JN, Simpkin AJ, O’Keeffe LM, Wade KH, Houtepen LC, Relton CL (2018). Epigenetic gestational age acceleration: a prospective cohort study investigating associations with familial sociodemographic and birth characteristics. Clin Epigenetics.

[30] Ross KM, Carroll JE, Horvath S, Hobel CJ, Coussons-Read ME, Dunkel-Schetter C (2020). Epigenetic age and pregnancy outcomes: GrimAge acceleration is associated with shorter gestational length and lower birthweight. Clin Epigenetics.

[31] Suhag A, Berghella V (2013). Intrauterine growth restriction (IUGR): etiology and diagnosis. Curr Obstet Gynecol Rep.

[32] Sharma D, Shastri S, Sharma P (2016). Intrauterine growth restriction: antenatal and postnatal aspects. Clin Med Insights Pediatr.

[33] Joung KE, Lee J, Kim JH (2020). Long-term metabolic consequences of intrauterine growth restriction. Curr Pediatr Rep.

[34] Girchenko P, Lahti J, Czamara D, Knight AK, Jones MJ, Suarez A (2017). Associations between maternal risk factors of adverse pregnancy and birth outcomes and the offspring epigenetic clock of gestational age at birth. Clin Epigenetics.

[35] Tekola-Ayele F, Workalemahu T, Gorfu G, Shrestha D, Tycko B, Wapner R (2019). Sex differences in the associations of placental epigenetic aging with fetal growth. Aging.

[36] Tekola-Ayele F, Workalemahu T, Gorfu G, Shrestha D, Tycko B, Wapner R (2019). Sex differences in the associations of placental epigenetic aging with fetal growth. Aging.

[37] Van Lieshout RJ, McGowan PO, de Vega WC, Savoy CD, Morrison KM, Saigal S (2021). Extremely low birth weight and accelerated biological aging. Pediatrics.

[38] Yan W, Zhang Y, Wang L, Yang W, Li C, Wang L (2019). Maternal dietary glycaemic change during gestation influences insulin-related gene methylation in the placental tissue: a genome-wide methylation analysis. Genes Nutr.

[39] Geraghty AA, Sexton-Oates A, O’Brien EC, Alberdi G, Fransquet P, Saffery R (2018). A low glycaemic index diet in pregnancy induces DNA methylation variation in blood of newborns: results from the ROLO randomised controlled trial. Nutrients.

[40] Park J-H, Kim S-H, Lee MS, Kim M-S (2017). Epigenetic modification by dietary factors: Implications in metabolic syndrome. Mol Aspects Med.

[41] Slater-Jefferies JL, Lillycrop KA, Townsend PA, Torrens C, Hoile SP, Hanson MA (2011). Feeding a protein-restricted diet during pregnancy induces altered epigenetic regulation of peroxisomal proliferator-activated receptor-α in the heart of the offspring. J Dev Orig Health Dis.

[42] Vamecq J, Latruffe N (1999). Medical significance of peroxisome proliferator-activated receptors. Lancet.

[43] Tyagi S, Gupta P, Saini AS, Kaushal C, Sharma S (2011). The peroxisome proliferator-activated receptor: a family of nuclear receptors role in various diseases. J Adv Pharm Technol Res.

[44] Irvin MR, Aslibekyan S, Do A, Zhi D, Hidalgo B, Claas SA (2018). Metabolic and inflammatory biomarkers are associated with epigenetic aging acceleration estimates in the GOLDN study. Clin Epigenetics.

[45] Wesolowski SR, Kasmi KCE, Jonscher KR, Friedman JE (2017). Developmental origins of NAFLD: a womb with a clue. Nat Rev Gastroenterol Hepatol.

[46] Chu DM, Antony KM, Ma J, Prince AL, Showalter L, Moller M (2016). The early infant gut microbiome varies in association with a maternal high-fat diet. Genome Med.

[47] González-Becerra K, Ramos-Lopez O, Barrón-Cabrera E, Riezu-Boj J, Milagro F, Martínez-López E (2019). Fatty acids, epigenetic mechanisms and chronic diseases: a systematic review. Lipids Health Dis.

[48] Burdge GC, Lillycrop KA (2014). Fatty acids and epigenetics. Curr Opin Clin Nutr Metab Care.

[49] • Koemel NA, Senior AM, Dissanayake HU, Ross J, McMullan RL, Kong Y, et al. Maternal dietary fatty acid composition and newborn epigenetic aging–a geometric framework approach. Am J Clin Nutr. 2021;115(1):118–27. 10.1093/ajcn/nqab318. **Demonstrates a complex nonlinear and interactive relationship between fatty acids and newborn epigenetic aging and potential protective effect of ω-3 fatty acids.** 10.1093/ajcn/nqab318 34591100

[50] Clare CE, Brassington AH, Kwong WY, Sinclair KD (2019). One-carbon metabolism: linking nutritional biochemistry to epigenetic programming of long-term development. Annu Rev Anim Biosci.

[51] Joubert BR, Herman T, Felix JF, Bohlin J, Ligthart S, Beckett E (2016). Maternal plasma folate impacts differential DNA methylation in an epigenome-wide meta-analysis of newborns. Nat Commun.

[52] Green R, Allen LH, Bjørke-Monsen A-L, Brito A, Guéant J-L, Miller JW (2017). Vitamin B 12 deficiency. Nat Rev Dis Primers.

[53] Sae-Lee C, Corsi S, Barrow TM, Kuhnle GGC, Bollati V, Mathers JC (2018). Dietary intervention modifies DNA methylation age assessed by the epigenetic clock. Mol Nutr Food Res.

[54] Monasso GS, Küpers LK, Jaddoe VWV, Heil SG, Felix JF (2021). Associations of circulating folate, vitamin B12 and homocysteine concentrations in early pregnancy and cord blood with epigenetic gestational age: the Generation R Study. Clin Epigenetics.

[55] Xue J, Schoenrock SA, Valdar W, Tarantino LM, Ideraabdullah FY (2016). Maternal vitamin D depletion alters DNA methylation at imprinted loci in multiple generations. Clin Epigenetics.

[56] Zhu H, Bhagatwala J, Huang Y, Pollock NK, Parikh S, Raed A (2016). Race/ethnicity-specific association of vitamin D and global DNA methylation: cross-sectional and interventional findings. PloS One.

[57] Chen L, Wagner CL, Dong Y, Wang X, Shary JR, Huang Y (2020). Effects of maternal vitamin D3 supplementation on offspring epigenetic clock of gestational age at birth: a post-hoc analysis of a randomized controlled trial. Epigenetics.

[58] Arora I, Sharma M, Sun LY, Tollefsbol TO (2020). The epigenetic link between polyphenols aging and age-related diseases. Genes.

[59] Vega CC, Reyes-Castro LA, Rodríguez-González GL, Bautista CJ, Vázquez-Martínez M, Larrea F (2016). Resveratrol partially prevents oxidative stress and metabolic dysfunction in pregnant rats fed a low protein diet and their offspring. J Physiol.

[60] Li Y, Liu H, Zhang L, Yang Y, Lin Y, Zhuo Y (2020). Maternal dietary fiber composition during gestation induces changes in offspring antioxidative capacity inflammatory response and gut microbiota in a sow model. Int J Mol Sci.

[61] Calatayud M, Koren O, Collado MC (2019). Maternal microbiome and metabolic health program microbiome development and health of the offspring. Trends Endocrinol Metab.

[62] Berni Canani R, Di Costanzo M, Leone L (2012). The epigenetic effects of butyrate: potential therapeutic implications for clinical practice. Clin Epigenetics.

[63] Vähämiko S, Laiho A, Lund R, Isolauri E, Salminen S, Laitinen K (2019). The impact of probiotic supplementation during pregnancy on DNA methylation of obesity-related genes in mothers and their children. Eur J Nutr.

[64] Wallace J, Gohir W, Sloboda D (2016). The impact of early life gut colonization on metabolic and obesogenic outcomes: what have animal models shown us?. J Dev Orig Health Dis.

[65] Wang M, Monaco MH, Donovan SM (2016). Impact of early gut microbiota on immune and metabolic development and function. Semin Fetal Neonatal Med.

[66] Indrio F, Martini S, Francavilla R, Corvaglia L, Cristofori F, Mastrolia SA (2017). Epigenetic matters: the link between early nutrition, microbiome, and long-term health development. Front Pediatr.

[67] Lu AT, Quach A, Wilson JG, Reiner AP, Aviv A, Raj K (2019). DNA methylation GrimAge strongly predicts lifespan and healthspan. Aging.

[68] Kim Y, Huan T, Joehanes R, McKeown NM, Horvath S, Levy D (2021). Higher diet quality relates to decelerated epigenetic aging. Am J Clin Nutr.

[69] Hartwig FP, de Mola CL, Davies NM, Victora CG, Relton CL (2017). Breastfeeding effects on DNA methylation in the offspring: a systematic literature review. PloS one..

[70] Pauwels S, Symons L, Vanautgaerden E-L, Ghosh M, Duca RC, Bekaert B (2019). The influence of the duration of breastfeeding on the infant’s metabolic epigenome. Nutrients.

[71] • Mallisetty Y, Mukherjee N, Jiang Y, Chen S, Ewart S, Arshad SH, et al. Epigenome-wide association of infant feeding and changes in DNA methylation from birth to 10 years. Nutrients. 2021;13(1):99. **First epigenome-wide comparison of breastfed to formula-fed infants from 0 to 10 years. This analysis revealed that DNA methylation patterns for specific genes differ based depending on whether they were breastfed or formula fed.** 10.3390/nu13010099 PMC7824231 33396735

[72] Verduci E, Banderali G, Barberi S, Radaelli G, Lops A, Betti F (2014). Epigenetic effects of human breast milk. Nutrients.

[73] Antonakou A, Skenderi KP, Chiou A, Anastasiou CA, Bakoula C, Matalas A-L (2013). Breast milk fat concentration and fatty acid pattern during the first six months in exclusively breastfeeding Greek women. Eur J Nutr.

[74] Innis SM (2014). Impact of maternal diet on human milk composition and neurological development of infants. Am J Clin Nutr.

[75] Simopoulos AP (2016). An increase in the omega-6/omega-3 fatty acid ratio increases the risk for obesity. Nutrients.

[76] Pannaraj PS, Li F, Cerini C, Bender JM, Yang S, Rollie A (2017). Association between breast milk bacterial communities and establishment and development of the infant gut microbiome. JAMA Pediatr.

[77] Babakobi MD, Reshef L, Gihaz S, Belgorodsky B, Fishman A, Bujanover Y (2020). Effect of maternal diet and milk lipid composition on the infant gut and maternal milk microbiomes. Nutrients.

[78] Paul B, Barnes S, Demark-Wahnefried W, Morrow C, Salvador C, Skibola C (2015). Influences of diet and the gut microbiome on epigenetic modulation in cancer and other diseases. Clin Epigenetics.

[79] Simpkin AJ, Hemani G, Suderman M, Gaunt TR, Lyttleton O, Mcardle WL (2015). Prenatal and early life influences on epigenetic age in children: a study of mother–offspring pairs from two cohort studies. Hum Mol Genet.

[80] Keikha M, Bahreynian M, Saleki M, Kelishadi R (2017). Macro-and micronutrients of human milk composition: are they related to maternal diet?. A comprehensive systematic review Breastfeed Med.

[81] Anderson CM, Gillespie SL, Thiele DK, Ralph JL, Ohm JE (2018). Effects of maternal vitamin D supplementation on the maternal and infant epigenome. Breastfeed Med.

[82] Christian P, Smith ER, Lee SE, Vargas AJ, Bremer AA, Raiten DJ (2021). The need to study human milk as a biological system. Am J Clin Nutr.

[83] Gensous N, Garagnani P, Santoro A, Giuliani C, Ostan R, Fabbri C (2020). One-year Mediterranean diet promotes epigenetic rejuvenation with country- and sex-specific effects: a pilot study from the NU-AGE project. GeroScience.

[84] • Fitzgerald KN, Hodges R, Hanes D, Stack E, Cheishvili D, Szyf M, et al. Potential reversal of epigenetic age using a diet and lifestyle intervention: a pilot randomized clinical trial. Aging. 2021;13(7):9419–32. 10.18632/aging.202913. **This pilot randomized control trial is the first study to demonstrate the reversal of epigenetic age in adults using a diet and lifestyle intervention.** 10.18632/aging.202913 PMC8064200 33844651

[85] Taylor RM, Smith R, Collins CE, Mossman D, Wong-Brown MW, Chan EC (2018). Methyl-donor and cofactor nutrient intakes in the first 2–3 years and global DNA methylation at age 4: a prospective cohort study. Nutrients.

[86] Montrose L, Ward TJ, Semmens EO, Cho YH, Brown B, Noonan CW (2017). Dietary intake is associated with respiratory health outcomes and DNA methylation in children with asthma. Allergy Asthma Clin Immunol.

[87] Marini S, Davis KA, Soare TW, Zhu Y, Suderman MJ, Simpkin AJ (2020). Adversity exposure during sensitive periods predicts accelerated epigenetic aging in children. Psychoneuroendocrinology.

[88] de Prado-Bert P, Ruiz-Arenas C, Vives-Usano M, Andrusaityte S, Cadiou S, Carracedo Á (2021). The early-life exposome and epigenetic age acceleration in children. Environ Int.

[89] Simpkin AJ, Howe LD, Tilling K, Gaunt TR, Lyttleton O, McArdle WL (2017). The epigenetic clock and physical development during childhood and adolescence: longitudinal analysis from a UK birth cohort. Int J Epidemiol.

[90] • Huang R-C, Lillycrop KA, Beilin LJ, Godfrey KM, Anderson D, Mori TA, et al. Epigenetic age acceleration in adolescence associates with BMI, inflammation, and risk score for middle age cardiovascular disease. J Clin Endocrinol Metab. 2019;104(7):3012–24. **This study revealed that higher body mass index, inflammation, and cardiovascular risk scores were associated with epigenetic aging in adolescents.** 10.1210/jc.2018-02076 PMC6555851 30785999

